# Innominate vein cannulation: easy but neglected technique

**DOI:** 10.15171/jcvtr.2018.32

**Published:** 2018-12-13

**Authors:** Mustafa Akbulut, Ozgur Arslan, Adnan Ak, Serpil Tas, Davut Cekmecelioglu, Mesut Sismanoglu, Altug Tuncer

**Affiliations:** Kosuyolu Kartal Heart Training and Research Hospital, Department of Cardiovascular Surgery, Istanbul, Turkey

**Keywords:** Innominate Vein, Cannulation Technique, Minimally Invasive

## Abstract

***Introduction:*** Our experience in minimally invasive procedures and improvement of graft technology enables easy and successful operation carried out even with complex thoracic aortic diseases from limited surgical area. However, it should be more than one incision or cannulation site for such intervention. We aimed to present our experience and results of 23 patients who has ascending aorta and aortic arch pathologies of which we operated with J-shaped partial sternotomy and innominate vein cannulation.

***Methods:*** From January 2014 to January 2016, 23 patients with aorta and aortic valve pathologies who underwent aortic surgery with J-shaped partial sternotomy and innominate vein cannulation included. Operation findings, cardiopulmonary bypass (CPB) values, postoperative results, surgical mortality and morbidity rates, late conversion to full sternotomy rates, ICU and hospital length of stay were evaluated.

***Results:*** The mean age of the patients was 53.7±12 (range 19-68) and 18 (78.2%) were males. Arcus aorta debranching applied to 4 patients (17.3%) and one of these procedures was frozen elephant trunk procedure (4.3%). Neither mortality nor cerebrovascular accident occurred. Mean CPB peak flow was 4.6±0.4 L/min, mean flow index calculated as 2.01±0.38 L/min/m2 and there was no CPB problem intraoperatively. Innominate vein ligation was carried out in 5 patients but no complication was seen except one who had left arm swelling treated with elevation.

***Conclusion:*** Innominate vein cannulation with J-shaped partial sternotomy is a reliable and easily applicable method providing effective utilization of limited operative field not only in ascending aorta and aortic arch operations but also with the advancements of hybrid systems used in descending aorta pathologies.

## Introduction

Compared to the conventional approaches in cardiac surgery, minimally invasive procedures has grown in popularity due to the lower complication rates and positive impact on quality of life during early postoperative period.^1-3^

To achieve better clinical outcomes, in addition to implementing of a successful surgical procedure, the surgeon must overcome the technical challenges posed by the narrow operative field. Choosing of an appropriate cannulation technique will be the decisive factor at this point.

**Figure 1:**
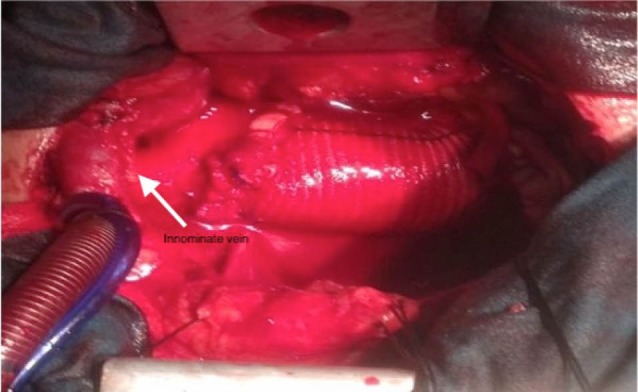
Placement of vein cannula from innominate vein to right atrium for supracoronary ascending aortic replacement.

In this article, we aimed to present our experience and results of 23 patients, of which we performed J-shaped partial sternotomy and innominate vein cannulation for ascending aorta and aortic arch pathologies (Figure 1).

## Materials and Methods

### Patient profile

A total of 23 patients with aorta and aortic valve pathologies who underwent aortic surgery with J-shaped partial sternotomy and innominate vein cannulation between January 2014 and January 2016 were included in the study. Data were collected prospectively and analyzed retrospectively. The mean age of the patients was 53.7±12 (range 19-68) and 18 (78.2%) were men. Demographic characteristics of the patients are shown in the Table 1.

**Table 1:** The characteristics of the patients

**Variable**	
Age, mean±SD/range	53.7±12/19-68
Male, No. (%)	18 (78.2%)
BMI (kg/m^2^), mean±SD	28.3±4.1
EF, mean±SD	60.2±7.6
Preoperative risk factors (%)	
Prior MI	4 (17.3%)
DM	18 (78.2%)
HTN	7 (30.4%)
Dialysis	1 (4.3%)
COPD	10 (43.4%)
CVA	0 (0%)
PVD	0 (0%)
CHF	1 (4.3%)
Patient characteristics, No (%)	
Severe AS/bicuspid aorta	5 (21.7%)/3 (13.0%)
Severe AR	6 (26.0%)
Chronic type I aortic dissection	1 (4.3%)
Marfan syndrome	2 (8.6%)

BMI; body mass index, MI; myocardial infarction, DM; Diabetes mellitus, COPD; Chronic obstructive pulmonary disease, HTN; Hypertension, CVA; Cerebrovascular accident, PVD; Peripheral vascular disease, CHF; Congestive heart failure, EF; Ejection Fraction, SD; Standard deviation, AS; Aortic stenosis, AR; Aortic regurgitation

Operation findings, cardiopulmonary bypass (CPB) values, postoperative results, surgical mortality and morbidity, late conversion to full sternotomy rates, ICU and hospital length of stay were evaluated.

### Definitions

Mortality corresponds to the first 30-day mortality. Preoperative and postoperative contrast enhanced thoracoabdominal computed tomography was used for the diagnosis of aortic pathology, preoperative planning and follow-up of patients. Patients who have a previous pulmonary disease diagnosis or pulmonary function tests with FEV1 <30% and FEV1/FVC <50% were accepted to have chronic obstructive pulmonary disease (COPD). Cerebrovascular events that occurred more than 72 hours ago, correspond to cerebrovascular disease definition.

### Patient selection

All patients were evaluated preoperatively with computed tomographic angiography (CT-A) for diagnostic purposes and determination of surgical indication. CT-A also provided valuable information about the level and anatomical suitability of the aortic root for the partial sternotomy procedure. Transthoracic echocardiography was used for the diagnosis of any valvular pathology and in patients older than 40 years of age coronary angiographic evaluation was carried out for any possible coronary artery disease. In one patient with chronic type-1 aortic dissection, myocardial perfusion assessment was done with coronary CT-A.

### Surgical technique

In all patients full monitoring was provided with central venous and arterial catheterizations and cerebral pulse oximetry. A cerebrospinal fluid drainage catheter was placed for pressure monitoring in the patient with chronic type-1 aortic dissection. Following the preparation of right subclavian artery, a J-shaped partial sternotomy was done through a limited skin incision with the ending zone at the level of fourth intercostal space. After the placement of pericardial traction stitches, ascending aorta and branches of aortic arch were prepared. Following the systemic heparinization, subclavian cannulation was done with a CalMed 18 Fr cannula. A Carpentier 24/29 Fr two-stage bi-caval venous cannula extending to inferior cava was placed at the proximal section of innominate vein where it joins superior vena cava and then cardiopulmonary bypass was initiated. In 5 patients with aortic arch involvement, innominate vein was divided to obtain both effective usage of the operative field and better manipulation of aortic arch branches. Venting cannula was inserted through right superior pulmonary vein. Following the aortic cross clamping, aortotomy was performed and cardiac arrest was achieved through administration of antegrade warm blood cardioplegia with an ostial cannula. For myocardial protection, intermittent antegrade cardioplegia was preferred.

We used tubular Dacron grafts for ascending aorta replacement and trousers Dacron grafts for debranching process. Prosthetic valves were chosen in patients that went through Bentall and Wheat procedures, except 3 patients who received Mitroflow Valsalva Conduit graft (Sorin group) for Bentall operation. In Frozen Elephant Trunk procedure 28 mm x 150 mm E-Vita Open Plus grafts (JOTEC^®^ GmbH, Germany) were used. Distal aortic anastomoses were made with unilateral antegrade selective cerebral perfusion at 28oC of body temperature after cross clamping of innominate artery. In case of left-sided oxygen saturation decrease in cerebral oximetry, left carotid artery cannulation was done and bilateral antegrade selective cerebral perfusion was initiated.

Surgical information and distribution of various operative techniques that were applied are shown in Table 2.

**Table 2:** Operative techniques and surgical information

**Procedures Performed**	**No. (%)**
Ascending aorta replacement	
Alone	4 (17.3)
Hemiarch replacement	5 (21.7)
Total Arc replacement	3 (13.0)
Bentall de Bono procedure	
Alone	5 (21.7)
Hemiarch replacement	3 (13.0)
Total Arc replacement	1 (4.3)
Wheat procedure	2 (8.6)
Operation Time (min)	Mean ±SD
TPT	131.3±49.3
CCT	79.9±31.5
ASCP	18.8±15.6

FET; frozen elephant trunk, TPT; total perfusion time, CCT; Cross clamp time, ASCP; antegrade selective cerebral perfusion, SD; Standard deviation.

## Results

Mean cross-clamp and CPB times were 79.9±31.5 and 131.3±49.3 minutes, respectively. All procedures were performed with antegrade selective cerebral perfusion at moderate hypothermia. Mean cerebral perfusion time was 18.8±15.6 minutes. None of the patients required a late conversion to full sternotomy. In 5 patients, innominate vein was divided after cannulation to obtain better exposure of aortic arch branches.

No hemodynamic problems were experienced during CBP. Flow parameters are shown in Table 3.

**Table 3:** Flow Characteristics

**BSA (m** ^2^ **)**	**Weight (kg)**	**Peak flow (L/min)**	**Flow Index (L/min/m2)**	**CVP**
**Mean±SD**	**Mean±SD**	**Mean±SD**	**Range**	**Mean±SD**
1.96±0.16	82.9±12.7	4.6±0.4	2.01±0.38	9.3±1.2

BSA: Body surface area, CVP: Central venous pressure, SD: Standard deviation

Postoperative outcomes are specified in Table 4. In-hospital mortality was not observed. None of the patients required revision surgery due to postoperative bleeding or cardiac tamponade. Only one patient with diabetes mellitus having a body mass index (BMI) of 35.6 kg/m^2^ was re-operated for sternal dehiscence. Also none of the patients experienced a neurological or a pulmonary complication. Only one in 5 patients whose innominate vein has been divided, had swelling of the left arm that regressed with elevation before discharging from the hospital. We have not seen any vein complication such as bleeding, need for innominate vein ligation, vein thrombosis and upper arm edema etc.

**Table 4:** Postoperative Outcomes

**Variable**	
Mortality, No. (%)	0 (0)
Cerebrovascular accident, No. (%)	0 (0)
Reoperation, No. (%)	1 (4.3)
Pulmonary complications, No. (%)	0 (0)
Renal insufficiency, No. (%)	0 (0)
Wound infection, No. (%)	0 (0)
Postoperative drainage (Mean) (mL)	491.3±94.9
ICU stay (Mean ±SD, min-max day)	1.2±0.41 (1-2)
Hospital stay (Mean ±SD, min-max day)	6.6±1.9 (4-11)

Pulmonary complications; >3 days of ventilator support or tracheostomy, Renal insufficiency; postoperative creatinine level >2.5 or temporary/permanent dialysis requirement, ICU; Intensive care unit, SD; Standard deviation, min; minimum, max; maximum.

All patients had gone through CT-A and Echocardiographic evaluation before discharge and there were no pathologies on the images.

### Statistical reviews

IBM SPSS Statistics 22 (IBM SPSS, Turkey) programs were used for statistical analysis when assessing the results obtained in this study. Descriptive statistical methods (mean, standard deviation, frequency) were used while data of the study were analyzing.

## Discussion

Although median sternotomy with its proven reliability is the standard approach method for aortic valve and ascending aorta pathologies, in recent years minimally invasive methods such as right partial-thoracotomy or various complex partial sternotomy procedures have been taking over in search to lower surgical trauma and complications.^4-6^

Those minimally invasive methods have comparably shorter ventilation times, lower transfusion rates, lesser post-operative pain, lower sternal dehiscence and wound infection rates, lower atrial fibrillation incidence, earlier mobilization and recovery periods leading to shorter hospitalization times.^1,2,5,7^

Minithoracotomy and partial-sternotomy have similar post-operative results comparing to median sternotomy. However, they have longer CPB and cross-clamp times. Working in a deeper surgical field requires the use of specially-designed surgical instruments.^8^ J-shaped^9,10^ or T-shaped partial sternotomies provide better exposure in ascending aorta, aortic arch and aortic root operations. Nevertheless difficulty in fixation of the 3 fragments of sternum in T-shaped sternotomy makes the process more risky in terms of sternal dehiscence.^5^

In minimally invasive surgery, to ensure the effective use of the limited surgical field, various cannulation techniques were described.^11^ Albeit central arterial cannulation is more physiological,^12^ in aortic pathologies including the aortic arch, its application is rather difficult. Therefore peripheral alternatives, such as axillary or femoral arteries are preferred. Femoral artery cannulation increases the risk of cerebrovascular event due to retrograde emboli especially in elderly patients with peripheral artery disease.13 During ascending aorta and aortic arch surgery with conventional median sternotomy, we perform open distal anastomosis with selective antegrade cerebral perfusion routinely due to its safety. Therefore we prefer the right axillary artery cannulation in our clinic.

Femoral vein, right atrium, percutaneous internal jugular vein or superior vena cava are the common sites for venous cannulation. Femoral vein cannulation requires an additional incision and has its own complications. There is also a possibility of inadequate flow that would necessitate the insertion of an extra cannula through superior vena cava or right atrium resulting in narrowing of operative field.11 During right atrial cannulation, passing out the venous cannula through a subxyphoidal skin incision might be difficult in patients with stretched or deeply placed right atrium.^14^

IJV cannulation, achieved by the insertion of small-diameter cannulas either with Doppler guide or Seldinger technique, proves to be a successful method providing adequate flow rates. However it might disrupt IJV valve leading to thrombus formation and respiratory brain syndrome. Although there are reports that this situation may have no clinical significance, it is still controversial.^15,16^ Furthermore according to Maltepe et al 16% of the cases with inadequate flow, required vacuum assist.^17^ In addition, Vaughan et al indicated that vacuum assisted venous return systems are not suitable for every patient, but applicable to patients with large BSA only.^18^

According to our observation of 788 complex aortic disease cases that we have operated with conventional median sternotomy between 2006 and 2014, we believe that division and cannulation of innominate vein is an alternative technique that can be used routinely. Being larger than IJV, its cannulation does not cause any valvular pathology and provides adequate flow rates with standard two-stage femoral cannulas, eliminating the requirement of a special cannula. Moreover without the necessity of an additional incision or a secondary venous cannula, more effective use of the limited operative field can be achieved.

Even if there is a laceration of innominate vein due to tractions and dissections during surgery or if accessing the branches of aortic arch is essential, in such cases division of innominate vein is convenient.^19^ Interruption of venous return may result in swelling of the left upper extremity and cerebral edema leading to neurological complications, both of which regress with elevation therapy. In our study we observed left arm swelling in only one patient, which returned to normal with elevation and the patient was discharged without any problem.

Although it was a retrospective study with small number of patients, we believe that innominate vein cannulation technique is successful if it is applied systematically in accordance with the conventional CBP principles.

Innominate vein cannulation with J-shaped partial sternotomy is a reliable and an easily applicable method enabling effective utilization of narrow operative field not only in ascending aorta and aortic arch operations but with the advancements of hybrid systems also in descending aorta pathologies.

## Ethical approval

This study was approved by the local ethics committee (No. 2018.7/3-127).

## Competing interests

There are no financial or other relations that could lead to a conflict of interest. There are any non-financial competing interests (political, personal, religious, ideological, academic, intellectual, commercial or any other) to declare in relation to this manuscript.
